# High Income Protects Whites but Not African Americans against Risk of Depression

**DOI:** 10.3390/healthcare6020037

**Published:** 2018-04-23

**Authors:** Shervin Assari

**Affiliations:** 1 Center for Research on Ethnicity, Culture, and Health (CRECH), School of Public Health, University of Michigan, Ann Arbor, MI 48104, USA; assari@umich.edu; Tel.: +1-734-363-2678; 2 Department of Psychiatry, University of Michigan, 4250 Plymouth Rd., Ann Arbor, MI 48109-2700, USA

**Keywords:** socioeconomic status, depression, major depressive disorder, ethnic health disparities, race, African Americans

## Abstract

*Background:* Built on the Blacks’ diminished return theory, defined as smaller effects of socioeconomic status (SES) on a wide range of health outcomes for African Americans compared to Whites, the current study compared African Americans and Whites for the association between household income and risk of lifetime, 12-month, and 30-day major depressive disorder (MDD). *Methods:* For the current cross-sectional study, we used data from the Collaborative Psychiatric Epidemiology Surveys (CPES), 2001–2003. With a nationally representative sampling, CPES included 4746 non-Hispanic African Americans and 7587 non-Hispanic Whites. The dependent variables were lifetime, 12-month, and 30-day MDD, measured using Composite International Diagnostic Interview (CIDI). The independent variable was household income. Age, gender, education, chronic medical conditions, and obesity were covariates. Race was the focal moderator. Logistic regression models were used to test the protective effects of household income against MDD in the overall sample and also by race. *Results:* In the overall sample, household income was inversely associated with the risk of 12-month and 30-day MDD. We found a significant interaction between race and household income on 12-month and 30-day MDD, suggesting a smaller protective effect of household income against MDD for African Americans compared to Whites. *Conclusion:* In line with the Blacks’ diminished return theory, household income better protects Whites than African Americans against MDD. The contribution of diminished return of SES as an underlying mechanism behind racial disparities in health in the United States is often overlooked. Additional research is needed on why and how SES resources generate smaller health gain among minority groups.

## 1. Introduction

Longitudinal and cross-sectional studies have strongly established the protective effects of socioeconomic status (SES) on population health [1,2,3,4,5,6]. SES indicators such education [7], employment [8,9], and income [1,4,5] protect individuals against morbidity [10] and mortality [11,12,13]. Income has protective effects against risk of depression [14].

However, population sub-groups do not similarly gain health from their SES indicators [15,16,17]. Some of the sociodemographic factors that alter the effects of SES include age [18], gender [3,19,20,21,22], race [19,20,21], and their intersections [22]. This is in line with the Blacks’ “diminished return” theory, suggesting that the protective effect of SES on health of populations is systemically smaller for African Americans in comparison to Whites [15,16,21]. Education [20], employment [23], and income [24] better reduce mortality and morbidity of the socially privileged than the socially disadvantaged group.

Research that shows SES effects are conditional by race [25,26] suggest that it is race and SES not race or SES that cause racial disparities [15,16]. If it is race and SES not race or SES, then SES does not fully explain the effects of race, and for the elimination of racial disparities in health, more needs to be done than merely eliminating racial disparities in SES [15,16]. That is, the elimination of SES disparities will not fully eliminate the racial disparities in health.

Regarding the effects of high SES on major depressive disorder (MDD), a meta-analysis showed that the prevalence, incidence, and persistence of MDD is lower in high-SES individuals compared to low-SES individuals [27]. However, individual studies have shown mixed results regarding the protective effects of SES indicators against risk of MDD [19,22,28,29]. Studies have suggested that the protective effects of SES indicators such as education and income against MDD and depressive symptoms may be larger for Whites than non-Whites [19,22]. In line with this literature, some research has documented an increase in the risk of depression in high-SES African Americans [22,28].

### Aims

The current study compared African Americans and Whites for the association between household income and lifetime, 12-month, and 30-day MDD.

## 2. Methods

### 2.1. Design and Setting

With a cross-sectional design, the current study used data from the Collaborative Psychiatric Epidemiology Surveys (CPES), 2001–2003. The CPES was conducted by the University of Michigan (UM, Ann Arbor, MI, USA). Although the CPES methods have been described in detail elsewhere [29], we briefly summarize the study methodology here.

CPES is composed of three national surveys: (1) the National Comorbidity Survey- Replication (NCS-R) [30], (2) the National Latino and Asian American Study (NLAAS) [31], and (3) the National Study of American Life (NSAL) [29]. The CPES data were collected by the University of Michigan (UM) Institute for Social Research (ISR), Ann Arbor.

### 2.2. Sampling

White and African American participants were recruited using the CPES core sampling. Core sampling of the CPES was a multistage stratified area probability sample that recruited a nationally representative household sample. All participants were adults (18 years of age and older). Participants were recruited from households in the coterminous 48 states. The sample was limited to individuals who were able to conduct an interview in English. This study did not include any institutionalized individuals. Thus, being in prisons, jails, nursing homes, and medical facilities were exclusion criteria [29]. African Americans and Whites in the CPES were selected from large cities, other urban areas, or rural areas [29]. The analysis for the current study included a total of 4746 non-Hispanic African Americans and 7587 non-Hispanic Whites.

### 2.3. Ethics

The CPES study protocol was approved by the University of Michigan (UM) Institutional Review Board (IRB # B03-00004038-R1). Informed written consent was received from all participants. Data were kept anonymous. Participants were financially compensated for their time. Publicly available CPES data were downloaded from Interuniversity Consortium for Political and Social Research (ICPSR https://www.icpsr.umich.edu), located at the University of Michigan Institute for Social Research.

### 2.4. Data Collection

CPES collected data using structured interviews (survey questionnaires). Most of the data were collected using computer-assisted face-to-face interviews. Telephone interviews were only used for the remaining data collection. Interviews lasted between two hours on average. The overall response rate of the CPES is 69%.

### 2.5. Measures

#### 2.5.1. Independent Variable

Household income was self-reported. Income was treated as a continuous measure in this study. To increase interpretability of the income coefficients, we divided income by USD 10,000. So, our income coefficients reflect the effect of a USD 10,000 increase in income on odds of MDD.

#### 2.5.2. Dependent Variable

Major Depressive Disorder (MDD). The presence of MDD (lifetime, 12-month, and 30-day) was evaluated using the World Mental Health (WMH) Composite International Diagnostic Interview (CIDI). The CIDI can be administered by trained interviewers who are not clinicians. Participants were assessed for meeting the DSM criteria for MDD. CIDI is frequently used for African Americans and Whites [32,33,34,35,36].

#### 2.5.3. Covariates

Covariates in this study included demographic characteristics (age and gender), health (chronic medical conditions and obesity), and socioeconomic status (education). Age was operationalized as a continuous variable. Gender was conceptualized as a dichotomous variable (male 1 vs. female 0). The socioeconomic covariate included education, which was measured as an ordinal variable with the following four categories: (1) less than 11 years, (2) 12 years, (3) between 13 and 15 years, and (4) 16 years or more. Education was operationalized as a categorical variable [37]. Chronic medical conditions and obesity were health covariates. Participants indicated whether or not they were ever told by a doctor or health professional that they had chronic medical conditions, including heart diseases, hypertension, chronic lung disease, asthma, diabetes, peptic ulcer, epilepsy, and cancer. Chronic medical conditions were defined as the number of chronic medical conditions, with a potential range from 0 to 8 [38,39,40]. Obesity was defined as having a body mass index (BMI) equal to or larger than 30. BMI was calculated using participants’ self-reported height and weight. The use of self-reported height and weight in the calculation of BMI has been validated [41,42].

#### 2.5.4. Moderator

*Race*. Race was self-identified in the CPSE [43,44,45,46]. African-Americans were defined as Blacks without any ancestral ties to the Caribbean. Race was treated as a dichotomous variable, with Whites being the reference category. (African Americans = 1 vs. Whites = 0). All African Americans and Whites entered in this analysis were non-Hispanic.

### 2.6. Statistical Analysis

#### 2.6.1. Weights

To accommodate the CPES’s sampling weight, which was due to the multi-stage sampling design of the NCS-R, NSAL, and NLAAS, Stata 13.0 (Stata Corp., College Station, TX, USA) was applied for all our data analysis. This approach will consider applying the CPES sampling weights. We used Taylor series linearization to re-estimate our standard errors. To perform our subsample analyses, we applied sub-pop survey commands in Stata.

#### 2.6.2. Analytical Plan

For descriptive purposes, we used mean (SE) and proportions (relative frequency). Bivariate analyses included independent sample *t*-test, Pearson Chi square, and Spearman correlation tests in the pooled sample and by race. For multivariable analysis, we used four logistic regression models. From independent sample *t*-test and Pearson Chi square tests, we only reported *p*-values. From Spearman correlation tests, we reported *rho* values. Adjusted odds ratios (OR), 95% confidence intervals (CIs), and *p*-values were reported. In our logistic regression models, we used household income as the independent variable, MDD (lifetime, 12-month, and 30-day) as the dependent variable, and socio-demographics as covariates. Race was the focal moderator. The first two logistic regression models were estimated in the pooled sample composed of both African Americans and Whites. *Model 1* did not include race by household income interaction. *Model 2* included the race by household income interaction term. Subsequently, we estimated race-specific logistic regression models. *Model 3* was estimated for Whites and *Model 4* was calculated for African Americans.

## 3. Results

### 3.1. Descriptive Statistics

Table 1 provides a summary of the descriptive statistics in the overall sample and by race. African Americans had lower education and household income in comparison to Whites. African Americans had lower odds of MDD than Whites (Table 1).

### 3.2. Bivariate Correlations

Table 2 presents the results of bivariate correlations in the pooled sample and by race. Household income showed negative correlation with 12-month and 30-day MDD in the pooled sample and White, but not African Americans (Table 2).

### 3.3. Logistic Regressions in the Overall Sample

Table 3 presents the results of three sets of logistic regression models in the pooled sample. Both models have household income as the independent variable, and lifetime, 12-month, and 30-day MDD as the dependent variables. *Model 1* only included the main effects. *Model 2* also included the race by household income interaction term. *Model 1* showed that high household income was associated with lower odds of MDD above and beyond the covariates. *Model 2* also showed an interaction between race and household income, suggesting that the protective effects of household income against 12-month and 30-day MDD are smaller for African Americans relative to Whites (Table 3).

### 3.4. Logistic Regressions by Race

Table 4 provides a summary of the results of two logistic regression models specific to Whites and African Americans. *Model 3* showed that in Whites, high household income was associated with lower odds of 12-month and 30-day MDD. *Model 4* showed that in African Americans, household income was not associated with odds of 12-month or 30-day MDD (Table 4).

## 4. Discussion

Built on the Blacks’ diminished return theory [15,16], the current study aimed to explore racial variation in the association between household income and 12-month and 30-day MDD. Our findings showed that while higher household income is associated with lower risk of 12-month and 30-day MDD overall, this health gain is disproportionate and unequal for Whites and African Americans.

By documenting the diminished mental health returns of household income for African Americans compared to Whites, our results support the Blacks’ diminished return theory [15,16]. Previously, smaller health effects of education, employment, and income were shown for physical health outcomes such as chronic disease and mortality in African Americans relative to Whites [20,21,23]. For instance, a recent study showed smaller protective effects of income on chronic medical conditions for African Americans compared to Whites [24]. The life expectancy gain that is expected to follow employment is smaller for African Americans compared to Whites [23]. Similar differential effects of education on health behaviors such as drinking between Whites and African Americans are shown [21]. In addition to economic resources, psychological assets such as affect, coping, sleep, self-rated health, and self-efficacy better serve the health of Whites than African Americans [47,48,49,50,51,52,53,54,55,56,57].

Blacks’ diminished return theory has attributed the diminished return of African Americans to the discrimination and structural racism that are embedded in the fabric of American society. American society functions in a way that constantly maximizes the benefits of Whites, with the unintended consequence of minimum health return for non-Whites including African Americans, Latinos, and Native Americans [15,16,23].

The results do not suggest that African Americans have a tendency to mismanage their economic resources such as income, or that Whites more effectively use their resources. Instead, we argue that the American social structure is failing the African American families, even high SES African American families who have successfully climbed the social ladder and earn high income. Regardless of their ambitions, the U.S. society makes them pay extra psychological costs for their social mobility. This is particularly shown in the studies showing poor mental health of high SES African Americans [19,58].

One major contribution of this study is to the theoretical models that are commonly used for health disparities research. In line with the Blacks’ diminished return theory, at least some of the disparities are not due to differential exposures, but differential effects of the very same exposures [15,16]. Unfortunately, differential effects of socioeconomic factors between African Americans and Whites is traditionally overlooked [20,21]. We believe that without an assumption that the protective effects of SES indicators are universal, researchers should systemically explore interactions between race and resources on health [15,16]. Another theoretical contribution of this study is that it may not be African Americans but Whites whose health declines more rapidly due to low SES. Several existing theories such as Double Jeopardy [28,59], Triple Jeopardy [60], Multiple Jeopardy [61], and Multiple Disadvantage [62] conceptualize minority status as a vulnerable status, meaning that minority populations’ health is more strongly dependent upon the presence or absence of very same risk or protective factor [61].

This is not the first study to show that race alters the health effects of SES indicators; however, most of this literature has focused on physical health outcomes such as mortality [63,64,65,66,67]. Relative to physical health outcomes [63,64,65,66,67], less is known about differential gains that follow SES indicators such as income on depression.

Similar to our findings, there is some research [19,21,63,64,68] showing that SES does not explain the effect of race on health, but interacts with race on health [39]. In this view, race limits how much individuals and groups can benefit from the very same SES resource [15,16]. These patterns will result in high levels of racial disparities in high levels of SES [39,60].

A greater differential effect of education is shown than the differential effects of income. This is partially because given the racism in the labor market and segregation, education is more likely than income to generate different outcomes [69,70,71,72]. Racial inequity in pay causes differential health gains of education and employment by race [60,71]. The current study shows that the same racial gap exists between Whites and African Americans in how they can use their income to gain mental health. The low mental health gain of high-SES African Americans may be because high-SES African Americans are commonly more discriminated against than low-SES African Americans [72].

### 4.1. Implications

Our findings have policy and public health implications. Policies and programs should also aim to reduce the diminished returns of African Americans as a strategy to eliminate health disparities [15,16]. Addressing health disparities should go beyond merely equalizing access to the SES resources or reducing extra risk factors in the lives of minorities [15,16].

The diminished health return of very same SES resources should be regarded as a major contributor of racial health disparities in the USA [73,74,75,76]. Policies that merely focus on a universal increase of all populations to SES indicators may widen the existing health disparities. Policy makers and program planners who are interested in eliminating the persisting racial health disparities in the USA should think beyond equalizing access to resources across populations. Tailored programs may be needed to ensure that all social groups equally benefit from the very same resources, regardless of their race and color. Policy and program evaluations should also consider the evaluation of the same policy or program by race, in order to understand how the very same policy is affecting population sub-groups, and whether our interventions are widening the existing gaps or not.

### 4.2. Limitations

Our study had its own limitations. Due to the cross-sectional design, the current study does not allow the establishment of causal associations between household income and CMC. Not only SES impacts mental health; poor mental health may interfere with productivity and income generation. Future research should also consider the risk of reverse causality between MDD and household. Another potential limitation of the current study is omitted confounders. We did not include several factors such as insurance, health care use, and history of encounters with the mental health care system. Similarly, this study was limited to individual characteristics. Future research should include higher-level SES indicators that reflect policy and communities for Whites and African Americans. Similar to other studies that compare racial groups for the effects of the same variable, differential validity may be a threat. MDD may be of different severity in Whites and African Americans [77].

## 5. Conclusions

To conclude, race was found to alter the magnitude of the association between household income and 12-month and 30-day MDD in the U.S. The effect of race is not just on the amount of SES indicators such as income, but also on how SES indicators impact the health of individuals. This may be because race is a very important social construct in the United States and shapes treatment by society and access to the opportunity structure.

## Figures and Tables

**Table 1:** Summary of descriptive statistics in the overall sample and by race.

Characteristics	All		Whites		African Americans	
	%	95% CI	%	95% CI	%	95% CI
Gender						
Men	52.00	50.72–53.28	51.59	50.09–53.10	54.68	53.34–56.02
Women	48.00	46.72–49.28	48.41	46.90–49.91	45.32	43.98–46.66
Education (≥12 years) *^,a^						
0–11 years	14.58	13.25–16.02	13.18	11.57–14.98	23.76	21.92–25.70
12 years	32.01	29.66–34.45	31.30	28.57–34.15	36.66	35.11–38.23
13–15 years	27.76	26.30–29.27	28.16	26.44–29.95	25.14	23.55–26.80
16 years+	25.65	23.33–28.12	27.36	24.63–30.28	14.44	12.74–16.33
Obesity *^,a^						
No	75.35	74.09–76.58	76.86	75.34–78.31	65.52	64.03–66.97
Yes	24.65	23.42–25.91	23.14	21.69–24.66	34.48	33.03–35.97
Lifetime Major Depressive Disorder *^,a^						
No	82.98	81.95–83.96	81.98	80.85–83.06	89.51	88.55–90.40
Yes	17.02	16.04–18.05	18.02	16.94–19.15	10.49	9.60–11.45
12-Month Major Depressive Disorder *^,a^						
No	93.14	92.59–93.66	92.93	92.31–93.51	94.52	93.74–95.21
Yes	6.86	6.34–7.41	7.07	6.49–7.69	5.48	4.79–6.26
30-Day Major Depressive Disorder *^,a^						
No	97.42	97.04–97.75	97.33	96.90–97.70	98.01	97.50–98.42
Yes	2.58	2.25–2.96	2.67	2.30–3.10	1.99	1.58–2.50
	**Mean**	**95% CI**	**Mean**	**95% CI**	**Mean**	**95% CI**
Age (years) *	43.09	42.37–43.82	44.65	43.64–45.65	40.78	38.66–42.90
Chronic medical conditions (CMC) *^,b^	0.68	0.65–0.71	0.73	0.70–0.77	0.83	0.73–0.93
Household Income (USD 10,000) *^,b^	5.99	5.69–6.28	6.34	5.92–6.76	4.40	3.78–5.02

* *p* < 0.05 for comparisons of Whites and African Americans. ^a^ Pearson Chi square. ^b^ Independent samples *t* test. CI: confidence interval.

**Table 2:** Spearman correlations in the pooled sample and by race.

Characteristics	1	2	3	4	5	6	7	8	9	10
**All**										
1 Race (African Americans)	1.00									
2 Gender (Women)	−0.05	1.00								
3 Age	−0.08	−0.04	1.00							
4 Chronic Medical Conditions	0.05	−0.01	0.37 *	1.00						
5 Obesity	0.09	−0.02	0.05	0.17 *	1.00					
6 Education (≥12 years)	−0.11 *	−0.02	−0.09	−0.12 *	−0.06	1.00				
7 Household Income (USD 10,000)	−0.14 *	0.12 *	−0.05	−0.12 *	−0.06	0.31 *	1.00			
8 Lifetime Major Depressive Disorder (MDD)	−0.07	−0.11 *	−0.03	0.01	0.03	0.05	0.01	1.00		
9 12-Month Major Depressive Disorder (MDD)	−0.02	−0.08	−0.08	0.03	0.01	0.00	−0.06	0.58 *	1.00	
10 30-Day Major Depressive Disorder (MDD)	−0.01	−0.05	−0.03	0.02	0.00	−0.02	−0.05	0.34 *	0.58 *	1.00
**Whites**										
2 Gender (Women)		1.00								
3 Age		−0.05	1.00							
4 Chronic Medical Conditions		−0.01	0.37 *	1.00						
5 Obesity		0.00	0.05	0.18 *	1.00					
6 Education (≥12 years)		−0.02	−0.10	−0.12 *	−0.06	1.00				
7 Household Income (USD 10,000)		0.12 *	−0.06	−0.12 *	−0.05	0.29 *	1.00			
8 Lifetime Major Depressive Disorder (MDD)		−0.12 *	−0.03	0.02	0.05	0.04	0.00	1.00		
9 12-Month Major Depressive Disorder (MDD)		−0.09	−0.08	0.03	0.02	−0.01	−0.07	0.57 *	1.00	
10 30-Day Major Depressive Disorder (MDD)		−0.05	−0.04	0.01	0.01	−0.02	−0.06	0.33 *	0.57 *	1.00
**African Americans**										
2 Gender (Women)		1.00								
3 Age		−0.01	1.00							
4 Chronic Medical Conditions		−0.02	0.36	1.00						
5 Obesity		−0.10 *	0.04	0.13 *	1.00					
6 Education (≥12 years)		−0.06	−0.09	−0.07	−0.03	1.00				
7 Household Income (USD 10,000)		0.12 *	−0.02	−0.13 *	−0.08	0.37 *	1.00			
8 Lifetime Major Depressive Disorder (MDD)		−0.06	−0.09	−0.03	0.00	0.04	−0.01	1.00		
9 12-Month Major Depressive Disorder (MDD)		−0.06	−0.09	0.02	−0.02	0.05	0.00	0.65 *	1.00	
10 30-Day Major Depressive Disorder (MDD)		−0.04	−0.02	0.02	−0.07	0.01	0.02	0.39 *	0.60 *	1.00

* *p* < 0.05.

**Table 3:** Summary of logistic regressions between household income and major depressive disorder (MDD) in the pooled sample.

Characteristics	*Model 1* Main Effects		*Model 2* Model 1 + Interactions	
	B	95% CI	B	95% CI
**Lifetime MDD**				
Race (African Americans)	0.57 ***	0.43–0.74	0.55 ***	0.40–0.74
Gender (Women)	0.60 ***	0.53–0.69	0.60 ***	0.53–0.69
Age	0.99 *	0.99–1.00	0.99 *	0.99–1.00
Chronic Medical Conditions	1.08 #	0.99–1.16	1.08 #	0.99–1.16
Obesity	1.28 ***	1.12–1.45	1.28 ***	1.12–1.45
Education (≥12 years)				
0–11 years				
12 years	1.03	0.77–1.39	1.03	0.77–1.39
13–15 years	1.15	0.94–1.40	1.15	0.94–1.40
16 years+	1.24 #	0.97–1.57	1.24 #	0.97–1.57
Household Income (USD 10,000)	1.00	0.99–1.02	1.00	0.99–1.02
Household Income (USD 10,000) × Race	-	-	1.01	0.97–1.05
Intercept	0.52 ***	0.39–0.69	0.52 ***	0.39–0.69
**12-Month MDD**				
Race (African Americans)	0.65 *	0.47–0.91	0.49 ***	0.34–0.73
Gender (Women)	0.56 ***	0.47–0.66	0.56 ***	0.47–0.66
Age	0.98 ***	0.97–0.98	0.98 ***	0.97–0.98
Chronic Medical Conditions	1.26 ***	1.10–1.44	1.26 ***	1.10–1.44
Obesity	1.14	0.92–1.41	1.14	0.92–1.41
Education (≥12 years)				
0–11 years				
12 years	0.72	0.49–1.07	0.72	0.49–1.07
13–15 years	0.81	0.62–1.04	0.80 #	0.62–1.04
16 years+	0.92	0.67–1.26	0.91	0.67–1.26
Household Income (USD 10,000)	0.96 **	0.93–0.99	0.96 **	0.93–0.99
Household Income (USD 10,000) × Race	-	-	1.07 *	1.00–1.14
Intercept	0.54 ***	0.39–0.75	0.55 ***	0.39–0.76
**30-Day MDD**				
Race (African Americans)	0.69	0.43–1.10	0.43	0.23–0.79
Gender (Women)	0.56 ***	0.41–0.77	0.56	0.41–0.77
Age	0.98 **	0.98–0.99	0.98	0.98–0.99
Chronic Medical Conditions	1.09	0.94–1.26	1.09	0.94–1.26
Obesity	1.42	0.78–2.62	1.43	0.78–2.62
Education (≥12 years)				
0–11 years				
12 years	0.55 *	0.31–0.97	0.55	0.31–0.97
13–15 years	0.55 *	0.33–0.92	0.55	0.33–0.92
16 years+	0.82	0.45–1.47	0.81	0.45–1.46
Household Income (USD 10,000)	0.94 *	0.89–0.99	0.94	0.89–0.99
Household Income (USD 10,000) × Race	-	-	1.12	1.00–1.26
Intercept	0.18 ***	0.10–0.34	0.19	0.10–0.35

# *p* < 0.1, * *p* < 0.05, ** *p* < 0.01, *** *p* < 0.001.

**Table 4:** Summary of logistic regressions between household income and major depressive disorder (MDD) in Whites and African Americans.

Characteristics	*Model 1* Whites		*Model 2* African Americans	
	B	95% CI	B	95% CI
**Lifetime MDD**				
Gender (Women)	0.60 ***	0.52–0.69	0.69 #	0.46–1.05
Age	0.99 *	0.99–1.00	0.99 #	0.98–1.00
Chronic Medical Conditions	1.08 #	0.99–1.17	1.03	0.82–1.30
Obesity	1.30 ***	1.14–1.47	0.84	0.55–1.27
Education (≥12 years)				
0–11 years				
12 years	1.02	0.75–1.38	1.48	0.70–3.13
13–15 years	1.13	0.92–1.39	1.56	0.73–3.33
16 years+	1.23 #	0.96–1.58	1.32	0.64–2.75
Household Income (USD 10,000)	1.00	0.99–1.02	1.00	0.96–1.04
Intercept	0.52 ***	0.38–0.69	0.30 ***	0.13–0.71
**12-Month MDD**				
Gender (Women)	0.51 *	0.28–0.92	0.56 ***	0.47–0.67
Age	0.98 *	0.96–0.99	0.98 ***	0.97–0.98
Chronic Medical Conditions	1.31	0.91–1.87	1.26 **	1.09–1.45
Obesity	0.76	0.43–1.34	1.16	0.93–1.44
Education (≥12 years)				
0–11 years				
12 years	1.55	0.61–3.95	0.70 #	0.47–1.06
13–15 years	1.57	0.59–4.20	0.78 #	0.60–1.03
16 years+	2.03	0.68–6.10	0.89	0.65–1.23
Household Income (USD 10,000)	1.01	0.93–1.09	0.96 **	0.93–0.99
Intercept	0.19 ***	0.06–0.56	0.55 ***	0.39–0.78
**30-Day MDD**				
Gender (Women)	0.56	0.41–0.78	0.40 *	0.18–0.93
Age	0.98	0.98–0.99	0.99	0.97–1.02
Chronic Medical Conditions	1.08	0.93–1.26	1.20	0.76–1.90
Obesity	1.49	0.81–2.76	0.31 *	0.11–0.87
Education (≥12 years)				
0–11 years				
12 years	0.54	0.30–0.97	0.92	0.31–2.77
13–15 years	0.52	0.31–0.89	1.83	0.52–6.43
16 years+	0.81	0.44–1.48	0.74	0.11–5.17
Household Income (USD 10,000)	0.94	0.89–0.99	1.03	0.93–1.14
Intercept	0.19	0.10–0.35	0.06 **	0.01–0.31

# *p* < 0.1, * *p* < 0.05, ** *p* < 0.01, *** *p* < 0.001.
